# Prevalence and risk factors of osteopenia in adults with short bowel syndrome: a retrospective longitudinal cohort study

**DOI:** 10.3389/fmed.2024.1422596

**Published:** 2024-12-11

**Authors:** Guangming Sun, Yufei Xia, Haoyang Wang, Yaqin Xiao, Li Zhang, Yupeng Zhang, Xuejin Gao, Xinying Wang

**Affiliations:** ^1^Department of General Surgery, Nanjing Jinling Hospital, Affiliated Hospital of Medical School, Nanjing University, Nanjing, China; ^2^Department of General Surgery, Jinling Hospital, School of Medicine, Southeast University, Nanjing, China

**Keywords:** short bowel syndrome, osteopenia, risk factor, prevalence, parenteral nutrition

## Abstract

**Background:**

Metabolic Bone Disease (MBD) is common in patients with short bowel syndrome (SBS). This study was to investigate the incidence and risk factors of osteopenia in adult SBS patients.

**Methods:**

Hospital records from January 2010 to December 2019 were used to identify all eligible patients. Logistic regression and a nomogram were used to analyze the data.

**Results:**

A total of 120 patients with SBS were included in this study, and 76 patients (63.3%) developed osteopenia during the 10-year observation period, The multivariate analysis using the logistic regression model demonstrated that age (OR = 1.070; 95%CI: 1.016–1.126, *p* = 0.010), female (OR = 5.098; 95%CI: 1.211–21.456, *p* = 0.026), tumor history (OR = 4.481; 95%CI: 1.125–17.854, *p* = 0.033), duration of SBS (OR = 1.0862; 95%CI: 1.022–1.103, *p* = 0.002) and remnant ileum (OR = 4.260; 95%CI: 1.210–15.002, *p* = 0.024) were independent risk factors for osteopenia in adults with SBS. The area under the curve (AUC) for the joint model (age, female, tumor history, duration of SBS, remnant ileum) was 0.848 and the corresponding sensitivity and specificity were 0.855 and 0.705, respectively. The C-index was 0.849 (95% CI: 0.778–0.917); thus, the predictions made by the model were close to the actual outcomes. In the decision curve analysis, the nomogram performed well and was feasible to make beneficial clinical decisions.

**Conclusion:**

This study reveals the high prevalence of osteopenia in SBS patients and highlights the importance of early identification and treatment of osteopenia. A nomogram may provide personalized prediction and guidance for medical intervention.

## Introduction

Short bowel syndrome (SBS) is a clinicopathological condition characterized by malabsorption and malnutrition following the surgical removal of a section of the small intestine. This condition leads to an inadequate capacity for the gut to absorb nutrients, often necessitating the reliance on parenteral nutrition (PN) for many SBS patients to maintain adequate nutrition supplement (1, 2). PN involves the continuous infusion of nutritional preparations through veins to meet the patient’s energy demands, making it a life-sustaining treatment for SBS patients (3). However, long-term use of PN or an inappropriate oral diet can lead to complications such as liver injury, kidney stones, and osteoporosis in SBS patients (4, 5).

The pathogenesis of metabolic bone disease (MBD) is likely associated with underlying conditions such as disease, malabsorption, inflammation, and certain medications like corticosteroids. PN treatment can also impact bone health (6), with factors like aluminum toxicity, vitamin D effects, and hypercalciuria. Micronutrient deficiencies or toxicities may contribute, but currently, there is no conclusive evidence linking abnormal micronutrient levels to MBD in patients on HPN (4). In a study conducted by ESPEN in 2002 with 165 patients, the prevalence of MBD was assessed using dual-energy X-ray absorptiometry (DXA). This study found that 138 patients (84%) met the criteria for osteopenia, while osteoporosis was present in 41% of the patients (7). The pathogenesis of osteopenia involves various complex factors, including those related to PN treatment, underlying diseases, and general lifestyle factors (8). Patients with SBS who have PN have an increased susceptibility to MBD, which is due to altered calcium and phosphate metabolism, vitamin D deficiency, and other nutritional deficiencies that impair bone formation and resorption. Osteopenia significantly increases the risk of fractures, impacting the quality of life for patients with SBS (9). Therefore, early identification and management of bone health issues are important for SBS patients.

This study aims to provide long-term, comprehensive data on the occurrence of osteopenia in a cohort of adult SBS patients and to identify risk factors for osteopenia early enough to prevent its development. Patients at risk of bone loss should be encouraged to take appropriate calcium and vitamin D supplements, increase their sunlight exposure, and consider milk supplementation as early as possible.

## Materials and methods

### Study design

The study utilized a database detailed in previous published studies (10) and was approved by the ethics committee of the Jinling Hospital, which followed the ethical guidelines outlined in the 1964 Helsinki Declaration and its subsequent revisions. From January 2010 to December 2019, the hospital records database in this study was used to retrospectively identify all patients with SBS who were admitted to the Intestinal Failure Clinical Nutrition Center. A remaining small intestine length of 200 cm or less was generally agreed upon as fitting the criteria for SBS (11). The study included patients with or without a history of PN use and waived informed consent since it was a retrospective study.

The patient inclusion criteria for this study were adult patients diagnosed with SBS who had regular bone mineral density (BMD) monitoring once a year. Exclusion criteria were: (1) patients aged over 80 years (Physical decline and coexistence of multiple underlying diseases), (2) prior history of bone disorders or bone loss before the diagnosis of SBS, (3) patients with active cancer or acquired immunodeficiency syndrome, (4) primary hyperparathyroidism, and (5) a history of long-term hormone use.

### Demographic and clinical variables

The study collected the following patient information based on the date of BMD assessment: sex, age, body weight, height, body mass index (BMI), drinking/smoking history, presence of diabetes mellitus, presence of hypertension, patient-generated subjective global assessment (PG-SGA) grade, nutritional risk screening 2002 (NRS-2002) score, and some laboratory blood examinations. Furthermore, the duration of SBS, causes of SBS, remaining length of the small intestine, and PN-dependence were also recorded.

### Diagnosis of osteopenia

BMD was measured at the double femur and lumbar spine using dual-energy X-ray absorptiometry (GE Medical Systems Lunar Prodigy). Before measurement, ensure DEXA is calibrated and its ray output stability checked. Explain the process to the patient to relieve anxiety and have them remove metal items to prevent measurement interference. Position the patient based on the measurement site. For the lumbar spine, the patient lies supine with knees bent. For the hip joint, the patient lies supine with legs straight and slightly internally rotated. Set parameters and scanning range according to the patient’s situation. After scanning, check data, calculate T- and Z-values against reference values, and generate a report with basic info, measurement values, interpretations, and clinical suggestions to assist doctors in decision-making. The WHO criterion was used to classify patients’ bone mass. For teenagers, men <50 years, and premenopausal women, a Z-score > −2.0 indicated that the patient’s BMD range was normal as compared to peers, while a Z-score ≤ −2.0 indicated that the patient had lower BMD range than his peers. For postmenopausal women and men older than 50, the T-score was recommended. A T-score > −1.0 indicated normal bone mass, while a T-score from −1.0 to −2.5 indicated reduced bone mass (osteopenia). A T-score ≤ −2.5 indicated severely reduced bone mass (osteoporosis) and severe osteoporosis could be diagnosed when there is low BMD with a clear evidence of a fragility fracture (8, 12). For convenience in statistical analysis, the study divided patients into the non-osteopenia group (Z-score > −2.0 or T-score > −1.0) and the osteopenia group (Z-score ≤ −2.0 or T-score ≤ −1.0).

### Statistical analysis

Continuous variables were presented as mean ± standard deviation, while categorical variables were expressed as absolute counts and percentages. To ensure normality, the Kolmogorov–Smirnov test was used. Proportions were compared using Chi-squared or Fisher’s exact tests, while the student t-test or Mann–Whitney U-test was used for continuous variables when appropriate. Logistic regression was used to assess potential risk factors associated with osteopenia in adults with SBS, and the results were presented in terms of odds ratio (OR) with 95% confidence intervals (CI). The R package ‘rms’ was used to generate nomograms of multivariable models, and the accuracy of the nomogram was evaluated using the receiver-operating characteristic (ROC) curve (13). In addition, the predictive performance of the model was validated by repeating bootstrap resampling 1,000 times. Model fitting, nomogram display, model validation, and prediction effectiveness evaluation were carried out using R 4.1.1 programming software.

All tests were two-sided, and statistical significance was set at *p* < 0.05. SPSS (version 23.0; IBM Corp, Armonk, NY, United States) was used for statistical analysis.

## Results

### Demographic and clinical characteristics of the study population

**Figure 1:**
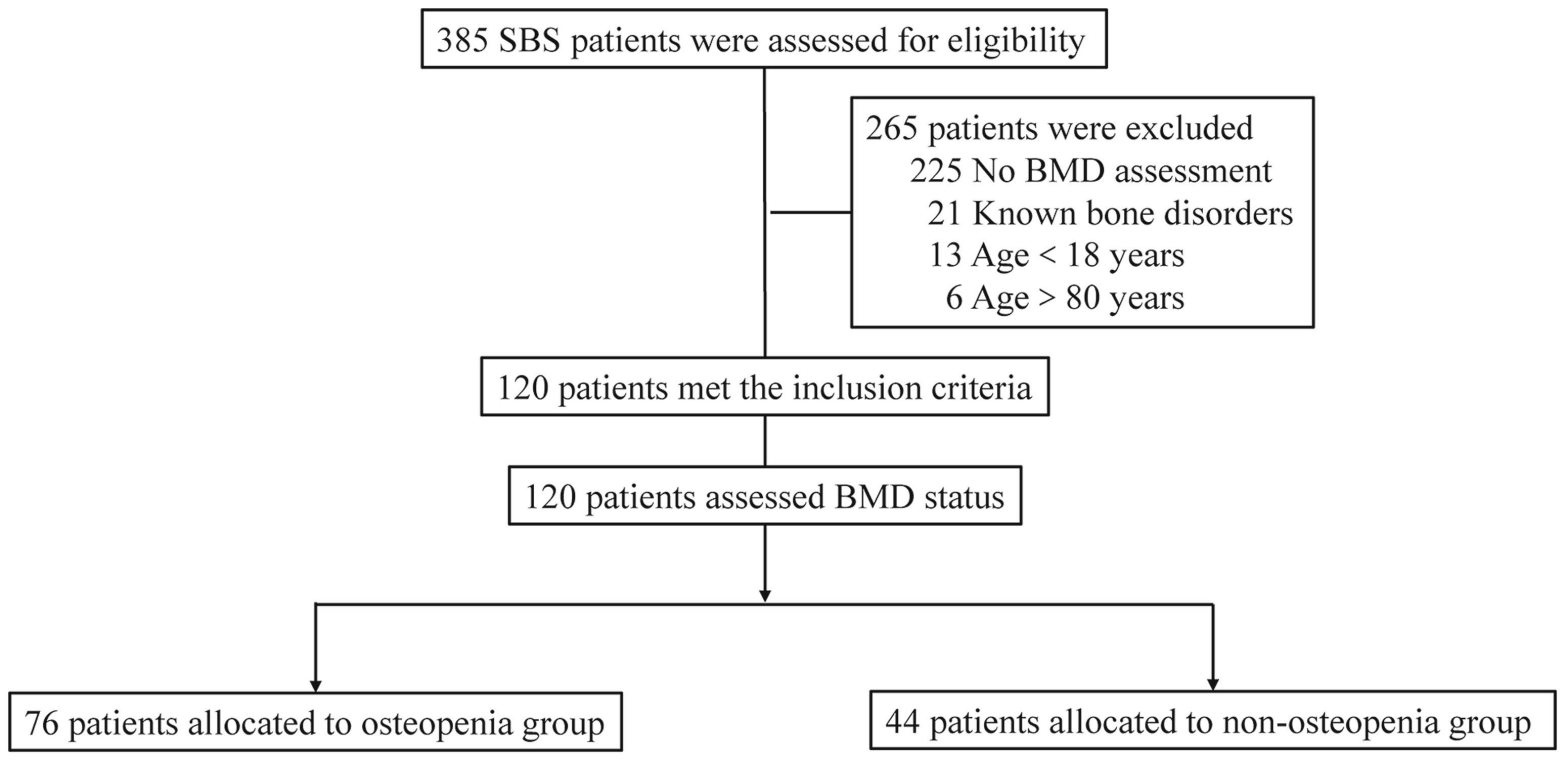
Flow diagram of patients with short bowel syndrome included in the study.

A total of 385 patients with SBS were assessed, of which 265 were excluded due to various reasons, including 225 without BMD assessments, 21 with known bone disorders, 13 aged under 18 years, and six aged over 80 years. Ultimately, 120 SBS patients were enrolled, with 76 patients in the osteopenia group and 44 in the non-osteopenia group (Figure 1).

The main demographic characters and the results of some laboratory tests for the included patients are shown in Table 1. The mean age of the patients was 53.6 ± 13.8 years with 68.3% of them being male. The differences in height and weight were as expected, and unexpectedly, we found no significant difference in BMI. Hypertension and diabetes were present in 23.5 and 5% of patients, respectively. Most patients (93.3%) suffered moderate to severe malnutrition (mean NRS-2002 score 4.1, 112 patients had PG-SGA grades B and C). A history of malignant tumors was reported by one-third of patients (30.8%), and just under half required HPN (40.8%). The median duration of SBS was 47 months. Nutritional indicators and relevant hematological results can be found in Supplementary Tables 1, 2, whereas underlying diseases in adult patients with SBS are shown in Supplementary Table 3.

**Table 1:** Demographic and laboratory tests for the study population.

Characteristic	ALL	Osteopenia	Non-osteopenia	*p* value
Number of patients (%)	120	76(63.3)	44(36.7)	
Age (years)	53.6 ± 13.8	57.2 ± 13.2	47.4 ± 12.7	**<0.001**
Sex (%)				**0.045**
Female	38(31.7)	29(38.2)	9(20.5)	
Male	82(68.3)	47(61.8)	35(79.5)	
Height (cm)	166.3 ± 7.9	164.7 ± 7.4	169.4 ± 8.1	**0.003**
Body weight (kg)	50.9 ± 11.4	48.8 ± 10.3	55.2 ± 12.5	**0.005**
BMI (kg/m^2^)	18.3 ± 3.3	17.9 ± 3.2	19.1 ± 3.3	0.091
Diabetes mellitus (%)	5(4.2)	3(4.1)	2(4.5)	1.000
Hypertension (%)	28(23.5)	17(22.7)	11(25)	0.772
Smoking (%)	17(17.3)	11(16.9)	6(18.2)	0.877
Alcohol (%)	20(20.4)	12(18.5)	8(24.2)	0.502
NRS2002 (%)	4.1 ± 1.1	4.0 ± 1.1	4.1 ± 1.0	0.875
PG-SGA (%)				0.388
A	7(5.9)	5(6.6)	2(4.7)	
B	60(50.4)	35(46.1)	25(58.1)	
C	52(43.7)	36(47.4)	16(37.2)	
Tumor (%)				**0.007**
Yes	37(30.8)	30(39.5)	7(15.9)	
No	83(69.2)	46(60.5)	37(84.1)	
PN dependence (%)				0.126
Yes	49(40.8)	35(46.1)	14(31.8)	
No	71(59.2)	41(53.9)	30(68.2)	
Duration of SBS (months)	47.0(39.0–70.0)	50.5(39.0–80.5)	42.5(38–51.8)	**0.008**

Values were presented as n (%), or mean ± SD, or median (first-to-third interquartile range). *p* < 0.05 is indicated by black bold. BMI, body mass index; PG-SGA, Patient-Generated Subjective Global Assessment; NRS-2002, nutritional risk screening 2002; PN, parenteral nutrition.

In the study of intestinal anatomy among SBS patients, the results showed that 44 patients (36.7%) had a jejunostomy (type I), 31 (25.8%) had a jejunocolic anastomosis (type II), and 45 (37.5%) had a jejunoileal anastomosis (type III). Of the patients in the osteopenia group, 34.2% had type III, while 31.8% of non-osteopenia group patients had type I. The mean length of the remaining small intestine was 93.2 ± 53.8 cm. In total, of 69 patients (57.5%) had a predominantly jejunum residual small intestine, 65% had an intact ileocecal valve, and 73.3% had colon integrity (Table 2).

**Table 2:** The intestinal anatomy of adult patients with SBS with and without osteopenia.

Characteristic	ALL	Osteopenia	Non-osteopenia	*p* value
Number of patients (%)	120	76(63.3)	44(36.7)	NA
Anatomy type (%)				0.608
Jejunostomy (type I)	44(36.7)	26(34.2)	18(40.9)	
Jejunocolic anastomosis (type II)	31(25.8)	19(25.0)	12(27.3)	
Jejunoileal anastomosis (type III)	45(37.5)	31(40.8)	14(31.8)	
Remaining small intestine length (cm)	93.2 ± 53.8	97.4 ± 59.5	85.9 ± 41.8	0.221
Intestine length type (%)				0.081
≤100	78(65)	45(59.2)	33(75)	
>100	42(35)	31(40.8)	11(25)	
Remaining small intestine type (%)				**0.001**
Jejunum predominantly	69(57.5)	35(46.1)	34(77.3)	
Ileum predominantly	51(42.5)	41(53.9)	10(22.7)	
Ileocecal valve intact (%)				0.874
Yes	78(65)	49(64.5)	29(65.9)	
No	42(35)	25(35.5)	15(34.1)	
Colon integrity (%)				0.458
Yes	88(73.3)	54(71.1)	34(77.3)	
No	32(26.7)	22(28.9)	10(22.7)	

Values were presented as n (%), or mean ± SD, or median (first-to-third interquartile range). *p* < 0.05 is indicated by black bold. SBS, short bowel syndrome.

### The prevalence of osteopenia in patients with SBS

Kaplan–Meier curve shows the cumulative incidence of osteopenia (Supplementary Figure 1). The incidence of osteopenia in SBS patients was 63.3% (76/120) over an observation period of 10 years. With increased duration of SBS, the incidence rate of bone disease goes up progressively. Patients were divided into the osteopenia group (*n* = 76) and the non-osteopenia group (*n* = 44) according to the BMD assessment. Significant differences were found in age, gender, tumor history, and duration of SBS between groups (*p* < 0.05; Table 1).The osteopenia group had a mean age of 57.2 ± 13.2 years with 29 (38.2%) of them being male and 30 (39.5%) with a tumor history. The average duration of SBS was 50.6 months according to our data. The two groups also differed significantly in nutritional and mineral indicators, such as albumin, HDL, GH, and zinc (*p* < 0.05; Supplementary Table 1). The remaining small intestine type, as a feature of intestinal anatomy, was significantly different between the groups; the proportion of patients with remnant ileum in the osteopenia group was higher than that in the non-osteopenia group [41 (53.9%) vs. 10 (22.7%), *p* = 0.001] (Table 2).

### The risk factors for osteopenia in SBS patients

We modeled the probability of developing osteopenia using univariable exact logistic regression and multivariable logistic regression. Univariable analysis showed a significant relationship between the risk of developing osteopenia and age, sex, tumor history, duration of SBS, remaining small intestine type, Zn, and Alb. These factors were further assessed in the multivariable model, where age (OR = 1.070; 95%CI: 1.016–1.126, *p* = 0.010), being female (OR = 5.098; 95%CI: 1.211–21.456, *p* = 0.026), tumor history (OR = 4.481; 95%CI: 1.125–17.854, *p* = 0.033), duration of SBS (OR = 1.0862; 95%CI: 1.022–1.103, *p* = 0.002), and remnant ileum (OR = 4.260; 95%CI: 1.210–15.002, *p* = 0.024) were identified as independent risk factors for developing osteopenia (Table 3). Supplementary Tables 4, 5 provide information about BMD*T- and Z-scores in adult patients with SBS with and without osteopenia, sorted by bone mass, sex, and age. Regardless of the age - and - gender - based grouping, significant differences in bone mineral density (BMD) are observed.

**Table 3:** Univariate and multivariate analysis of the independent variables associated with osteopenia in the entire population of patients with SBS study.

Independent variable	Univariable analysis		Multivariable analysis	
	OR(95% CI)	*p* value	OR(95% CI)	*p* value
Age (years)	1.058(1.026–1.092)	**<0.001**	1.070(1.016–1.126)	**0.010**
Sex Female/Male	2.400(1.009–5.707)	**0.048**	5.098(1.211–21.456)	**0.026**
Tumor Yes/No	3.447(1.361–8.734)	**0.009**	4.481(1.125–17.854)	**0.033**
Duration of SBS	1.028(1.009–1.047)	**0.003**	1.062 (1.022–1.103)	**0.002**
Ileocecal valve intact Yes/No	1.065(0.488–2.325)	0.874		
Colon in continuity Yes/No	1.385(0.585–3.280)	0.459		
Remaining small intestine type ileum/jejunum predominantly	3.983(1.725–9.198)	**0.001**	4.260(1.210–15.002)	**0.024**
PN dependence Yes/No	1.829(0.840–3.984)	0.128		
Intestine length type >100/≤100	0.484(0.213–1.100)	0.083		
GH	1.289(0.977–1.700)	0.072		
Zn	0.87(0.767–0.987)	**0.030**	0.974(0.818–1.160)	0.770
Alb	0.933(0.872–1.000)	**0.048**	0.974(0.865–1.097)	0.664
HDL	4.102(0.972–17.306)	0.055		

*p value <0.05 is indicated by black bold. SBS, short bowel syndrome; OR, odds ratio; CI, confidence interval. Zn, zinc; GH, growth hormone; Alb, albumin; HDL, high density lipoprotein.*

### Model development for the prediction of osteopenia in SBS patients

**Figure 2:**
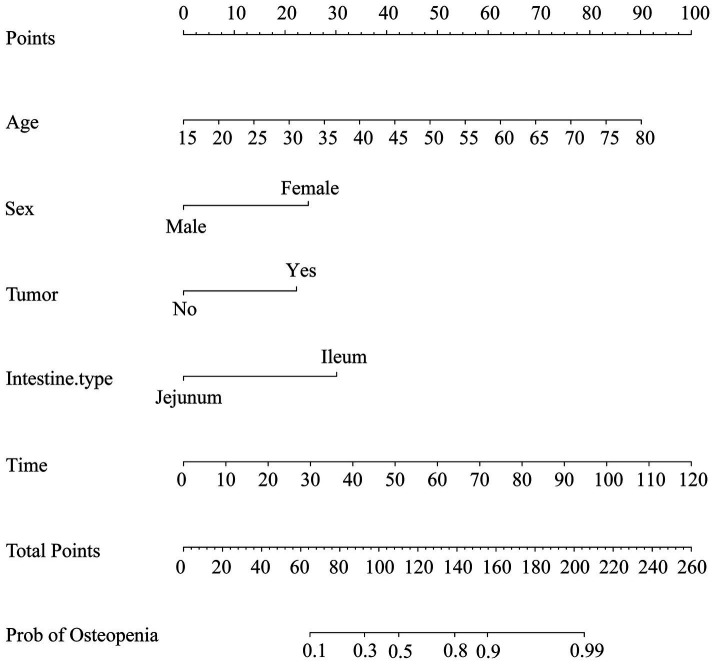
Nomogram for the prediction of osteopenia. As an example, an adult patient with SBS, 55 years old, scored 55 points; a female patient scored 25 points; a tumor history scored 22.5 points; the rest of the small intestine was mainly ileum, scored 30 points; 100 months history of SBS, scored 82.5 points; the total score was 215 points; therefore, the possibility of bone loss was greater than 99%.

**Figure 3:**
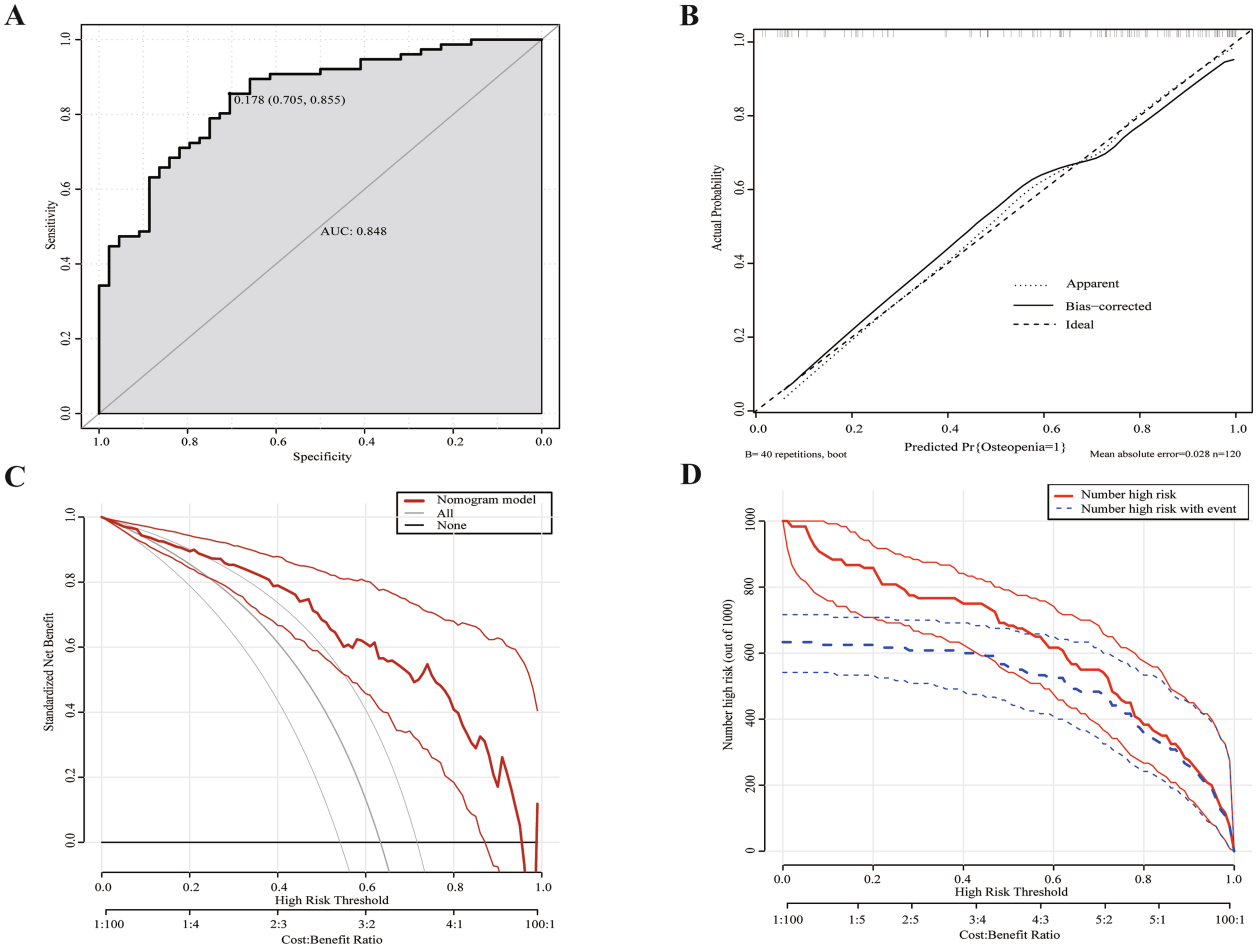
Evaluation of nomogram model. **(A)** ROC curve for the nomogram evaluation. The cut-off value was 0.178 with an area under the curve of 0.848. **(B)** Calibration curves of the nomogram. **(C)** DCA for the nomogram. Using multivariate combined nomographs to predict osteopenia has a higher net benefit than treating either no or all patients. **(D)** CIC for the nomogram. The red curve (the number of individuals at high risk) indicates the number of persons who are classified as positive (high risk) by the prediction model at each threshold probability; the blue curve (the number of individuals at high risk with osteopenia) is the number of true positives at each threshold probability. Two light colored lines represent a 95% confidence interval.

The logistic regression analysis results allowed us to create a joint model using all identified independent risk factors (age, being female, tumor history, duration of SBS, and remnant ileum) to predict osteopenia in patients with SBS. A nomogram illustrating the predictive variables and corresponding point scales is displayed in Figure 2 (14). Meanwhile, The ROC curve, calibration curve, decision curve analysis, and clinical impact curve for the nomogram are shown in Figure 3. The calibration of the model was assessed with calibration curves, which measure the relationship between the outcomes predicted by the model and the observed outcomes in the cohort. The calibration curve would lie on the diagonal 45-degree line in an ideal nomogram. The dashed line indicates the performance of an ideal nomogram, and the solid line indicates the performance of the present nomogram. The joint model’s area under the curve was 0.848, with corresponding sensitivity and specificity values of 0.855 and 0.705, respectively. We conducted a multiple bootstrap procedure (*n* = 1,000 bootstraps) to estimate the significance of these analyses, and the C-index was 0.848 (95% CI: 0.778–0.917). The decision curve analysis indicated that the nomogram was useful for making beneficial clinical decisions.

## Discussion

The present study is the first to investigate osteopenia in adult patients with short bowel syndrome (SBS). Through analysis of clinical data, several variables, including age, duration of SBS, and sex, were identified as impacting the loss of bone mass in individuals with intestinal failure (IF). Using these risk factors, a nomogram was developed to identify osteopenia as early as possible. This tool has the potential to improve the management and treatment of osteopenia in patients with SBS, particularly by enabling earlier intervention to prevent the development of more severe bone deficiencies.

According to a previous study, the frequency of bone loss in patients with an ostomy due to inflammatory bowel diseases was 29.4% and was attributed to an average age of 45 years old (15). However, our study showed a higher frequency of bone loss at 63.3%, with an average age of 53.6 years. In a multicenter study, 84% of patients receiving home parenteral nutrition (HPN) had bone disease according to the bone mineral density (BMD) T-score, and 62% according to the BMD Z-score, with half of these patients classified as having severe osteopenia (7). Patients with SBS at a rate of 40% also exhibited osteopenia. In these patients, osteopenia was associated with a tendency toward prolonged PN treatment compared to normal patients (11.58 vs. 2.39, *p* = 0.094) (16). Weaning rates from PN among individuals with IF range from 20 to 50% after 2 years (17). Our study found that 40.8% (49/120) of patients were PN-dependent, and among them, 46.1% (35/76) had osteopenia. A follow-up study of patients with IF showed no link between HPN and a decrease in BMD; instead, BMD was negatively correlated with being female and older age (18). Similarly, the results of our study showed that PN-dependency was not the primary influencing factor (*p* = 0.128). However, subsequent investigations involving comparatively large patient cohorts suggest that prolonged HPN is not necessarily linked to a reduction in BMD, and in certain instances, bone density may actually rise during HPN treatment (18).

A randomized controlled trial of growth hormone (GH) showed a significant increase in blood osteocalcin levels after 12 weeks of treatment (+62%; *p* < 0.05) (19) However, our study did not find a statistically significant difference. While GH may promote calcium absorption, bone development, and anabolic activity, it may not be the main factor influencing bone mass in patients with SBS. Instead, an imbalanced distribution of nutrients, particularly mineral insufficiency resulting from reduced dietary intake and impaired absorption of metallic elements, may contribute to increased bone loss and deterioration of bone microstructure in elderly patients. This could be the primary factor in the pathophysiological mechanism of osteopenia (20). Micronutrients such as iron, zinc, and copper play a significant role in the development and transformation of new bones, and their levels in the body can affect the synthesis of bone matrix and bone minerals (21). Our study showed no significant differences in minerals between the two groups, except for Zn (*p* = 0.03), which is an essential micronutrient for bone formation and maintenance of homeostasis. Imbalances in iron metabolism can increase osteoblast development and death, while inhibiting osteoblast proliferation and differentiation, leading to osteoporosis (22). However, our research revealed no such differences, which may be attributed to the poor nutritional status of our patients. The integrity of the colon is essential for compensatory absorption of triglycerides and fatty acids released from bacterial breakdown in patients with SBS, providing energy production (16). Our results showed no significant differences in BMD between SBS patients with or without colonic integrity, suggesting that while the colon may play a role in calorie clearance, it does not have a significant impact on BMD. Certain researchers hypothesize that ileectomy may contribute to bone loss, as this area is vital for the absorption of vitamin D and the synthesis of glucagon-like peptide 2 (GLP-2), which has been demonstrated to enhance bone mineral density (BMD) in certain patients (23). Moreover, long-term use of HPN may also lead to complications such as cholestasis, affecting the absorption of fat-soluble vitamins (such as vitamins A, D, E, and K). The application of antidiarrheal drugs may also affect the metabolism of nutrients such as calcium and other essential minerals in the body, thereby having a negative impact on bone density (5).

Risk factors for osteopenia in SBS patients can be identified by obtaining comprehensive bone health history, monitoring laboratory values, and conducting nutrition-focused physical examinations. Based on the results, it is advisable for all SBS patients to undergo DXA follow-ups every 1–3 years (24). When oral medication is needed for the prevention or treatment of osteoporosis, special consideration should be given to the high incidence of impaired gastrointestinal absorption. Direct injection is recommended for patients with impaired gastrointestinal absorption (25).

Despite the identification of independent risk factors for osteopenia in SBS patients and the provision of helpful aids for clinical decision-making, our study still has some limitations. Firstly, our sample population consists of older individuals, which may introduce bias as age itself is closely related to BMD. Additionally, sex also plays a role in osteopenia, particularly in post-menopausal women. Subgroup analyses by gender and age were not conducted, but the stability of our model was ensured through bootstrap sampling. The objective of this study was to identify risk factors for osteopenia in patients with SBS and provide data from China on this condition. Secondly, data on vitamin D (102/120) and parathyroid hormone (34/120) only partially available, there may be bias in the results, and we will continue to pay attention to the effect of PTH on BMD in the follow-up. Studies indicate that deficiencies in vitamin D impact calcium and phosphorus metabolism, bone mineralization, and the attainment of peak bone mass. Even slight deficiencies in vitamin D are linked to skeletal homeostasis and/or the inability to achieve peak bone mass (26, 27). But, when vitamin D less than <20 ng/mL was defined as deficiency, the percentage of vitamin D deficiency was 87% (89/102) in our study. By this standard, vitamin D insufficiency or deficiency is globally widespread, accounting for about 50 to 80 percent of the total population (28, 29). Surveys in Chinese cities at different latitudes have shown that vitamin D insufficiency or deficiency is common in the population (30, 31). Vitamin D is synthesized in the skin under ultraviolet radiation B (UVB) from sunlight or comes from food (natural and fortified). SBS patients are at high risk of vitamin D insufficiency/deficiency due to poor intake, absorption, and lack of UVB. They need more vitamin D supplements or sunlight (32). We tried to analyze that in the non-osteopenia group 13.79 ± 5.60 ng/mL, 13.24 ± 4.51 in Osteopenia group with a *p* value of 0.58, there was no significant difference between the two groups. Therefore, most patients have vitamin D deficiency, and vitamin D may not be a key factor in the reduction of bone mass in patients with short bowel syndrome, which may be more related to age, parenteral nutrition-related factors, and the condition of the intestinal tract itself. A prospective study by Shengxian fan (6) found vitamin D deficiency in 95% (57/60) of patients with short bowel syndrome. L. Ellegard et al. investigated 78 patients with intestinal failure vitamin D and bone mineral density, there was no significance association between vitamin D deficiency and low BMD (6, 33). We speculate that due to individual differences, some populations may have low detection values, but their vitamin D receptors on bone cells function well and can efficiently utilize limited vitamin D to regulate processes such as bone remodeling are still functional and can effectively utilize scarce vitamin D to regulate processes like bone remodeling (34). Genetic factors can promote traits that help keep bone density normal, even with low vitamin D levels. This can lessen the harmful effects of vitamin D deficiency on bone density (35). Furthermore, Mendelian randomization analysis also revealed no genetic causality between vitamin D and BMD (36). Finally, future studies involving larger sample sizes are required. Hence, timely detection of osteopenia in patients is crucial, and appropriate supplementation of trace elements based on the patient’s condition should be considered to prevent further deterioration. Intramuscular injection of vitamin D and zoledronic acid may also be considered as preventive measures. Furthermore, our aim is to gather more relevant data to validate the accuracy of our model and provide further valuable insights for SBS research.

## Conclusion

SBS in adult patients is associated with a significantly high risk of developing osteopenia. Independent predictors for osteopenia include the type of residual small intestine, age, sex, duration of SBS, and a history of tumors. In order to minimize the adverse effects of osteopenia, close monitoring and preventive interventions are recommended.

## Data Availability

The raw data supporting the conclusions of this article will be made available by the authors, without undue reservation.

## Supplementary material

SUPPLEMENTARY FIGURE 1 Kaplan–Meier plot showing adult patients with short bowel syndrome without osteopenia during the 10-year period.

## References

[1] Kitano K Schwartz DM Zhou H Gilpin SE Wojtkiewicz GR Ren X. Bioengineering of functional human induced pluripotent stem cell-derived intestinal grafts. Nat Commun. (2017) 8:765. doi: 10.1038/s41467-017-00779-y, PMID: 29018244 PMC5635127

[2] Iyer K DiBaise JK Rubio-Tapia A. AGA clinical practice update on Management of Short Bowel Syndrome: expert review. Clin Gastroenterol Hepatol Off Clin Pract J Am Gastroenterol Assoc. (2022) 20:2185–2194.e2. doi: 10.1016/j.cgh.2022.05.032, PMID: 35700884

[3] Cederholm T Barazzoni R Austin P. ESPEN guidelines on definitions and terminology of clinical nutrition. Clin Nutr Edinb Scotl. (2017) 36:49–64. doi: 10.1016/j.clnu.2016.09.004 27642056

[4] Cuerda C Pironi L Arends J Bozzetti F Gillanders L Jeppesen PB. ESPEN practical guideline: clinical nutrition in chronic intestinal failure. Clin Nutr Edinb Scotl. (2021) 40:5196–220. doi: 10.1016/j.clnu.2021.07.002 34479179

[5] Abi Nader E Lambe C Talbotec C Acramel A Pigneur B Goulet O. Metabolic bone disease in children with intestinal failure is not associated with the level of parenteral nutrition dependency. Clin Nutr. (2021) 40:1974–82. doi: 10.1016/j.clnu.2020.09.014 32977995

[6] Fan S Ni X Wang J Zhang Y Tao S Kong W. High prevalence of suboptimal vitamin D status and bone loss in adult short bowel syndrome even after weaning off parenteral nutrition. Nutr Clin Pract. (2017) 32:258–65. doi: 10.1177/0884533616665784 27589260

[7] Pironi L Labate AMM Pertkiewicz M Przedlacki J Tjellesen L Staun M. Prevalence of bone disease in patients on home parenteral nutrition. Clin Nutr Suppl. (2002) 21:289–96. doi: 10.1054/clnu.2002.0548, PMID: 12135588

[8] Napartivaumnuay N Gramlich L. The prevalence of vitamin D insufficiency and deficiency and their relationship with bone mineral density and fracture risk in adults receiving long-term home parenteral nutrition. Nutrients. (2017) 9:481. doi: 10.3390/nu9050481, PMID: 28489034 PMC5452211

[9] Consensus development conference. Diagnosis, prophylaxis, and treatment of osteoporosis. Am J Med. (1993) 94:646–50. doi: 10.1016/0002-9343(93)90218-e 8506892

[10] Gao X Zhang L Wang S Xiao Y Song D Zhou D. Prevalence, risk factors, and complications of Cholelithiasis in adults with short bowel syndrome: a longitudinal cohort study. Front Nutr. (2021) 8:762240. doi: 10.3389/fnut.2021.762240, PMID: 34912839 PMC8667726

[11] Pironi L. Definitions of intestinal failure and the short bowel syndrome. Best Pract Res Clin Gastroenterol. (2016) 30:173–85. doi: 10.1016/j.bpg.2016.02.011 27086884

[12] on behalf of the Scientific Advisory Board of the European Society for Clinical and Economic Aspects of Osteoporosis (ESCEO) and the Committees of Scientific Advisors and National Societies of the International Osteoporosis Foundation (IOF) Kanis JA Cooper C Rizzoli R Reginster JY. European guidance for the diagnosis and management of osteoporosis in postmenopausal women. Osteoporos Int. (2019) 30:3–44. doi: 10.1007/s00198-018-4704-5, PMID: 30324412 PMC7026233

[13] DeLong ER DeLong DM Clarke-Pearson DL. Comparing the areas under two or more correlated receiver operating characteristic curves: a nonparametric approach. Biometrics. (1988) 44:837–45. doi: 10.2307/2531595, PMID: 3203132

[14] Semenkovich TR Yan Y Subramanian M Meyers BF Kozower BD Nava R. A clinical nomogram for predicting node-positive disease in esophageal Cancer. Ann Surg. (2021) 273:e214–21. doi: 10.1097/SLA.0000000000003450, PMID: 31274650 PMC6940556

[15] Gupta S Wu X Moore T Shen B. Frequency, risk factors, and adverse sequelae of bone loss in patients with ostomy for inflammatory bowel diseases. Inflamm Bowel Dis. (2014) 20:259–64. doi: 10.1097/01.MIB.0000439065.92211.d3, PMID: 24378598

[16] Chiplunker AJ Chen L Levin MS Warner BW Davidson NO Rubin DC. Increased adiposity and reduced lean body mass in patients with short bowel syndrome. Dig Dis Sci. (2020) 65:3271–9. doi: 10.1007/s10620-019-06032-4 31907775 PMC7924810

[17] Parreiras-E-Silva LT de Araújo IM Elias J. Osteoporosis and hepatic steatosis: 2 closely related complications in short-bowel syndrome. JPEN J Parenter Enteral Nutr. (2020) 44:1271–9. doi: 10.1002/jpen.1802, PMID: 32048748

[18] Pironi L Tjellesen L de Francesco A Pertkiewicz M Morselli Labate AM Staun M. Bone mineral density in patients on home parenteral nutrition: a follow-up study. Clin Nutr Suppl. (2004) 23:1288–302. doi: 10.1016/j.clnu.2004.04.003, PMID: 15556251

[19] Tangpricha V Luo M Fernández-Estívariz C Gu LH Bazargan N Klapproth JM. Growth hormone favorably affects bone turnover and bone mineral density in patients with short bowel syndrome undergoing intestinal rehabilitation. JPEN J Parenter Enteral Nutr. (2006) 30:480–6. doi: 10.1177/0148607106030006480, PMID: 17047171

[20] Ceylan MN Akdas S Yazihan N. Is zinc an important trace element on bone-related diseases and complications? A Meta-analysis and systematic review from serum level, dietary intake, and supplementation aspects. Biol Trace Elem Res. (2021) 199:535–49. doi: 10.1007/s12011-020-02193-w 32451694

[21] Lin S Yang F Ling M Fan Y. Association between bone trace elements and osteoporosis in older adults: a cross-sectional study. Ther Adv Musculoskelet Dis. (2022) 14:1759720X221125984. doi: 10.1177/1759720X221125984, PMID: 36185074 PMC9523847

[22] Che J Yang J Zhao B Zhang G Wang L Peng S. The effect of abnormal Iron metabolism on osteoporosis. Biol Trace Elem Res. (2020) 195:353–65. doi: 10.1007/s12011-019-01867-4, PMID: 31473898

[23] Haderslev KV Jeppesen PB Hartmann B Thulesen J Sorensen HA Graff J. Short-term Administration of Glucagon-like Peptide-2. Effects on bone mineral density and markers of bone turnover in short-bowel patients with no Colon. Scand J Gastroenterol. (2002) 37:392–8. doi: 10.1080/003655202317316006, PMID: 11989828

[24] Seidner DL. Parenteral nutrition-associated metabolic bone disease. JPEN J Parenter Enteral Nutr. (2002) 26:S37–42. doi: 10.1177/014860710202600511, PMID: 12216719

[25] Davila J Konrad D. Metabolic complications of home parenteral nutrition. Nutr Clin Pract Off Publ Am Soc Parenter Enter Nutr. (2017) 32:753–68. doi: 10.1177/0884533617735089 29016233

[26] Campos DJ Boguszewski CL Funke VAM. Bone mineral density, vitamin D, and nutritional status of children submitted to hematopoietic stem cell transplantation. Nutr Burbank Los Angel Cty Calif. (2014) 30:654–9. doi: 10.1016/j.nut.2013.10.014 24613437

[27] Nygaard L Skallerup A Olesen SS Køhler M Vinter-Jensen L Kruse C. Osteoporosis in patients with intestinal insufficiency and intestinal failure: prevalence and clinical risk factors. Clin Nutr. (2018) 37:1654–60. doi: 10.1016/j.clnu.2017.07.018 28823627

[28] van Schoor N Lips P. Global overview of vitamin D status. Endocrinol Metab Clin N Am. (2017) 46:845–70. doi: 10.1016/j.ecl.2017.07.002, PMID: 29080639

[29] Holick MF Binkley NC Bischoff-Ferrari HA Gordon CM Hanley DA Heaney RP. Evaluation, treatment, and prevention of vitamin D deficiency: an Endocrine Society clinical practice guideline. J Clin Endocrinol Metab. (2011) 96:1911–30. doi: 10.1210/jc.2011-0385, PMID: 21646368

[30] Ning Z Song S Miao L Zhang P Wang X Liu J. High prevalence of vitamin D deficiency in urban health checkup population. Clin Nutr Edinb Scotl. (2016) 35:859–63. doi: 10.1016/j.clnu.2015.05.019 26093537

[31] Man PW van der Meer IM Lips P Middelkoop BJC. Vitamin D status and bone mineral density in the Chinese population: a review. Arch Osteoporos. (2016) 11:14. doi: 10.1007/s11657-016-0265-4, PMID: 27026017 PMC4819723

[32] Klampfer L. Vitamin D and colon cancer. World J Gastrointest Oncol. (2014) 6:430–7. doi: 10.4251/wjgo.v6.i11.430, PMID: 25400874 PMC4229786

[33] Ellegård L Kurlberg G Bosaeus I. High prevalence of vitamin D deficiency and osteoporosis in out-patients with intestinal failure. Clin Nutr Edinb Scotl. (2013) 32:983–7. doi: 10.1016/j.clnu.2013.02.005, PMID: 23481225

[34] Masuyama R Nakaya Y Katsumata S Kajita Y Uehara M. Dietary calcium and phosphorus ratio regulates bone mineralization and turnover in vitamin D receptor knockout mice by affecting intestinal calcium and phosphorus absorption. J Bone Miner Res. (2003) 18:1217–26. doi: 10.1359/jbmr.2003.18.7.1217 12854831

[35] Reid IR Bolland MJ Grey A. Effects of vitamin D supplements on bone mineral density: a systematic review and meta-analysis. Lancet. (2014) 383:146–55. doi: 10.1016/S0140-6736(13)61647-5 24119980

[36] Li S-S Gao L-H Zhang X-Y He J-W Fu W-Z Liu Y-J. Genetically low vitamin D levels, bone mineral density, and bone metabolism markers: a Mendelian randomisation study. Sci Rep. (2016) 6:33202. doi: 10.1038/srep33202, PMID: 27625044 PMC5021966

