# Supplementary figures and images for: Prevalence and risk factors of osteopenia in adults with short bowel syndrome: a retrospective longitudinal cohort study
Source: Front Med (Lausanne). 2024 Dec 11;11:1422596. doi: 10.3389/fmed.2024.1422596 (PMC11668563; doi:10.3389/fmed.2024.1422596)

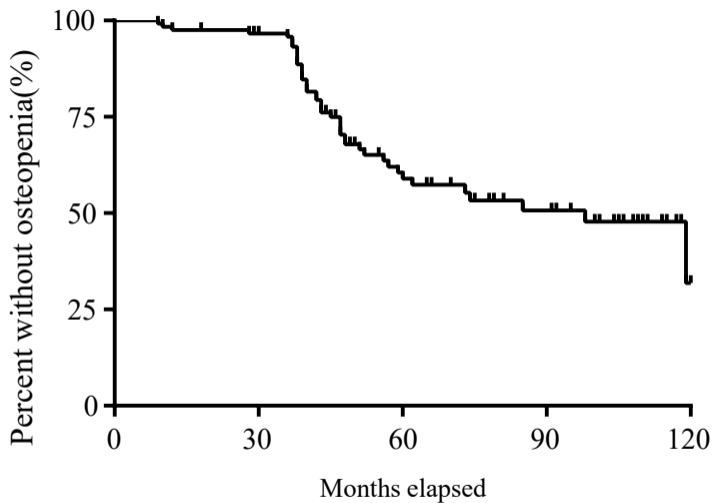

Number at risk

120

97

61

51

32

Supplement: SUPPLEMENTARY FIGURE 1 — Kaplan–Meier plot showing adult patients with short bowel syndrome without osteopenia during the 10-year period. [file Image_1.pdf]
