# Supplementary material for: Prevalence and risk factors of osteopenia in adults with short bowel syndrome: a retrospective longitudinal cohort study
Source: Front Med (Lausanne). 2024 Dec 11;11:1422596. doi: 10.3389/fmed.2024.1422596 (PMC11668563; doi:10.3389/fmed.2024.1422596)
Supplement: Supplementary file 2 [file Data_Sheet_1.docx]

**Supplementary table 1** | Nutritional and mineral indicators for the study population.

| Characteristic | ALL | Osteopenia | Non-osteopenia | *p* value |
| --- | --- | --- | --- | --- |
| **Laboratory tests** |  |  |  |  |
| Alb (g/L) | 36.0±5.9 | 35.2±5.5 | 37.5±6.3 | **0.045** |
| Prealbumin (mg/L) | 248.3±95.4 | 241.9±89.5 | 259.8±105.5 | 0.369 |
| Transferrin (g/L) | 1.9±0.9 | 1.8±0.94 | 2.0±0.94 | 0.218 |
| RBP (mg/L) | 36.6±16.7 | 36.6±18.5 | 36.6±13.4 | 0.996 |
| IGF-1 (μg/L) | 146.7±64.2 | 140.7±63.7 | 157.0±64.5 | 0.233 |
| HDL (mmol/L) | 0.8±0.3 | 0.8±0.4 | 0.7±0.2 | **0.033** |
| GH (μg/L) | 0.8(0.2-2.0) | 0.9(0.3-2.4) | 0.4(0.1-1.6) | **0.022** |
| Vitamin D (ng/mL) | 13.45±4.94 | 13.24±4.51 | 13.79± 5.60 | 0.58 |
| PTH (ng/L) | 43.01±7.48 | 44.43±8.09 | 42.05±7.08 | 0.36 |
| **Inorganic** |  |  |  |  |
| Calcium (mmol/L) | 2.1±0.2 | 2.1±0.2 | 2.2±0.2 | 0.370 |
| Zinc (μmol/L) | 13.3±3.6 | 12.7±3.5 | 14.4±3.6 | **0.026** |
| Copper (μmol/L) | 12.3(8.9-17.1) | 11.6(8.6-17.1) | 14.0(10.2-17.1) | 0.304 |
| Magnesium (mmol/L) | 0.8±0.1 | 0.8±0.2 | 0.8±0.1 | 0.642 |
| Phosphorus (mmol/L) | 1.1±0.3 | 1.1±0.3 | 1.1±0.3 | 0.970 |
| Potassium (mmol/L) | 4.0±0.6 | 4.0±0.6 | 4.1±0.5 | 0.463 |
| Chlorine (mmol/L) | 104.5±6.4 | 104.8±7.2 | 103.9±4.7 | 0.479 |
| Sodium (mmol/L) | 140.8(137-143) | 140.8(136-143) | 140.5(137.3-143) | 0.919 |
| Iron(μmol/L) | 13.3±8.8 | 13.4±9.4 | 13.2±7.6 | 0.904 |

Values were presented as n (%), or mean ± SD, or median (first-to-third interquartile range).

*p* < 0.05 is indicated by black bold. Alb, Albumin; RBP, retinol-binding protein; IGF-1, insulin-like growth factor 1; HDL, high density lipoprotein; GH, growth hormone; PTH, parathyroid hormone.

**Supplementary table 2** | Relevant hematological results for the study population.

| Characteristic | ALL | Osteopenia | Non-osteopenia | *p* value |
| --- | --- | --- | --- | --- |
| IL-6(ng/L) | 22.9±48.7 | 20.2±29.9 | 27.61±70.8 | 0.197 |
| CRP (mg/L) | 4.1(0.7-11.9) | 3.4(0.7-12.0) | 5.1(0.6-9.8) | 0.976 |
| TBIL (µmol/L) | 12.4(7.2-20.9) | 12.1(7.1-20.3) | 12.9(7.7-21.4) | 0.346 |
| DBIL (µmol/L) | 4.6(2.6-7.8) | 4.3(2.4-7.7) | 5.5(3.0-11.3) | 0.240 |
| ALT (U/L) | 27.2(16.3-43.8) | 27.0(17.1-40.3) | 28.2(16.0-50.5) | 0.363 |
| AST (U/L) | 24.0(17.0-33.8) | 24.0(17.0-31.7) | 24.7(16.9-36.5) | 0.977 |
| ALP (U/L) | 107.6±70.8 | 112.3±84.3 | 99.2±36.2 | 0.255 |
| γ-GT (U/L) | 41(19-91) | 42.0(19.0-80.0) | 36.0(18.5-128.0) | 0.906 |
| Triglyceride(mmol/L) | 1.4±0.9 | 1.3±0.9 | 1.5±1.0 | 0.354 |
| Cholesterol (mmol/L) | 3.0±1.1 | 2.9±1.0 | 3.0±1.2 | 0.724 |
| BUN (mmol/L) | 6.5(4.7-8.3) | 6.2(4.7-8.4) | 6.8(4.6-8.3) | 0.819 |
| Serum creatinine (mmol/L) | 83.1±76.9 | 74.7±62.4 | 97.7±96.0 | 0.114 |
| Hemoglobin (g/L) | 112.5±21.0 | 109.8±20.0 | 117.1±22.1 | 0.224 |
| PLT (×10^9^/L) | 166.8(128.7-228.3) | 171.0(128.7-240.8) | 161.2(129.3-197.8) | 0.204 |
| lymphocyte (×10^9^/L) | 1.2±0.6 | 1.2±0.6 | 1.3±0.6 | 0.398 |

Values were presented as n (%), or mean ± SD, or median (first-to-third interquartile range).

*p* < 0.05 is indicated by black bold. IL-6, Interleukin-6; CRP, C-reactive protein; TBIL, total bilirubin; DBIL, direct bilirubin; ALT, alanine aminotransferase; AST, aspartate aminotransferase; ALP, alkaline phosphatase; γ-GT, Gamma-glutamyl transferase; BUN, blood urea nitrogen, PLT, platelet.

**Supplementary table 3** | The underlying diseases in adult patients with SBS with and without osteopenia.

| Characteristic | ALL | Osteopenia | Non-osteopenia | *p* value |
| --- | --- | --- | --- | --- |
| Number of patients | 120 | 76 | 44 | **NA** |
| The etiology of SBS |  |  |  | 0.115 |
| Mesenteric ischemia | 47(39.2) | 27(35.5) | 20(45.5) |  |
| Surgical complications | 37(30.8) | 23(30.3) | 14(31.8) |  |
| Volvulus | 15(12.5) | 10(13.2) | 5(11.4) |  |
| Trauma | 4(3.3) | 1(1.3) | 3(6.8) |  |
| Crohn’ s disease | 3(2.5) | 2(2.6) | 1(2.3) |  |
| Radiation enteritis | 12(10.0) | 11(14.5) | 1(2.3) |  |
| Others | 2(1.7) | 2(2.6) | 0(0.0) |  |

Values were presented as n (%), SBS, short bowel syndrome.

**Supplementary table 4** | BMD* T- and Z-score in adult patients with SBS with and without Osteopenia.

| Patient groups | Lumbar spine | | | |  | Femoral neck | | | |
| --- | --- | --- | --- | --- | --- | --- | --- | --- | --- |
|  | No. of pts | BMD  (g/cm2) | T-score  (mean±SD) | Z-score  (mean±SD) |  | No. of pts | BMD  (g/cm2) | T-score  (mean±SD) | Z-score  (mean±SD) |
| Non-osteopenia | 44 | 1.12±0.13 | 0.17±1.07 | 0.81±0.99 |  | 44 | 0.96±0.13 | -0.03±0.96 | 0.64±1.03 |
| Osteopenia | 76 | 0.87±0.14 | -1.92±1.23 | -0.49±1.22 |  | 76 | 0.70±0.11 | -1.99±0.92 | -0.76±1.07 |
| Reduced | 44 | 0.93±0.12 | -1.39±0.92 | -0.23±1.18 |  | 44 | 0.77±0.08 | -1.46±0.71 | -0.50±1.07 |
| Severely reduced | 28 | 0.79±0.14 | -2.62±1.25 | -0.74±1.20 |  | 28 | 0.61±0.07 | -2.75±0.63 | -1.12±1.03 |
| Severely osteoporosis | 4 | 0.78±0.13 | -2.98±1.28 | -1.65±0.79 |  | 4 | 0.64±0.10 | -2.50±0.75 | -1.08±0.59 |

BMD = bone mineral density. T-score: number of standard deviations from mean BMD value of young sex-matched subjects.

No. of pts = Number of patients

Reduced = T-score below -1 SD but less than -2.5 SD; Severely reduced = T-score below -2.5SD;

Severely osteoporosis = T-score below -2.5SD with evidence of a fragility fracture

Z-score: number of standard deviations from mean BMD normal values corrected for sex and age.

Reduced = Z-score below -1SD but less than -2SD; Severely reduced = Z-score below -2SD.

**Supplementary table 5** | BMD* T- and Z-score in adult patients with SBS with and without Osteopenia, grouped by gender and age.

| Patient groups | Lumbar spine | | | |  | Femoral neck | | | |
| --- | --- | --- | --- | --- | --- | --- | --- | --- | --- |
| Sex/age | No. of pts | BMD  (g/cm^2^) | T-score (mean±SD) | Z-score  (mean±SD) |  | No. of pts | BMD  (g/cm^2^) | T-score  (mean±SD) | Z-score  (mean±SD) |
| Males | 82 | 0.99±0.19 | -0.87±1.54 | -0.01±1.42 |  | 82 | 0.83±0.18 | -1.09±1.38 | -0.26±1.37 |
| Females | 38 | 0.89±0.16 | -1.81±1.36 | -0.01±1.01 |  | 38 | 0.73±0.13 | -1.66±1.12 | -0.24±0.97 |
| *p* value |  | 0.040 | 0.002 | 0.994 |  |  | 0.001 | 0.028 | 0.878 |
| ＜50 | 45 | 1.04±0.19 | -0.54±1.53 | 0.02±1.42 |  | 45 | 0.86±0.17 | -0.78±1.35 | -0.33±1.38 |
| ≥50 | 75 | 0.91±0.17 | -1.53±1.44 | -0.03±1.22 |  | 75 | 0.76±0.16 | -1.57±1.23 | -0.20±1.18 |
| *p* value |  | ＜0.001 | 0.001 | 0.837 |  |  | 0.003 | 0.001 | 0.559 |

BMD = bone mineral density. No. of pts = Number of patients.

T-score: number of standard deviations from mean BMD value of young sex-matched subjects.

Z-score: number of standard deviations from mean BMD normal values corrected for sex and age.
